# Expert versus generalist inserters for peripheral intravenous catheter insertion: a pilot randomised controlled trial

**DOI:** 10.1186/s13063-018-2946-3

**Published:** 2018-10-17

**Authors:** Nicole Marsh, Joan Webster, Emily Larsen, Jodie Genzel, Marie Cooke, Gabor Mihala, Sue Cadigan, Claire M Rickard

**Affiliations:** 1 Royal Brisbane and Women’s Hospital, Herston, QLD Australia; 2 School of Nursing and Midwifery, Griffith University, Nathan, QLD Australia; 3 Alliance for Vascular Access Teaching and Research Group, Menzies Health Institute Queensland, Brisbane, QLD Australia; 4 School of Nursing, Queensland University of Technology, Kelvin Grove, QLD Australia; 5 School of Medicine, Griffith University, Gold Coast, QLD Australia; 6 Centre for Applied Health Economics, Menzies Health Institute Queensland, Brisbane, QLD Australia

**Keywords:** Intravenous, Vascular access devices, Randomised controlled trial, Phlebitis

## Abstract

**Background:**

Peripheral intravenous catheters (PVCs) are essential invasive devices, with 2 billion PVCs sold each year. The comparative efficacy of expert versus generalist inserter models for successful PVC insertion and subsequent reliable vascular access is unknown.

**Methods:**

A single-centre, parallel-group, pilot randomised controlled trial (RCT) of 138 medical/surgical patients was conducted in a large tertiary hospital in Australia to compare PVC insertion by (1) a vascular access specialist (VAS) or (2) any nursing or medical clinician (generalist model). The primary outcome was the feasibility of a larger RCT as established by predetermined criteria (eligibility, recruitment, retention, protocol adherence). Secondary outcomes were PVC failure: phlebitis, infiltration/extravasation, occlusion, accidental removal or partial dislodgement, local infection or catheter-related bloodstream infection; dwell time; insertion success, insertion attempts; patient satisfaction; and procedural cost-effectiveness.

**Results:**

Feasibility outcomes were achieved: 92% of screened patients were eligible; two patients refused participation; there was no attrition or missing outcome data. PVC failure was higher with generalists (27/50, 54%) than with VASs (33/69, 48%) (228 versus 217 per 1000 PVC days; incidence rate ratio 1.05, 95% confidence interval 0.61–1.80). There were no local or PVC-related infections in either group. All PVCs (*n* = 69) were successfully inserted in the VAS group. In the generalist group, 19 (28%) patients did not have a PVC inserted. There were inadequate data available for the cost-effectiveness analysis, but the mean insertion procedure time was 2 min in the VAS group and 11 min in the generalist group. Overall satisfaction with the PVC was measured on an 11-point scale (0 = not satisfied and 10 = satisfied) and was higher in the VAS group (*n* = 43; median = 7) compared to the generalist group (*n* = 20; median = 4.5). The multivariable model identified medical diagnosis and bed-bound status as being significantly associated with higher PVC failure, and securement with additional non-sterile tape was significantly associated with lower PVC failure.

**Conclusion:**

This pilot trial confirmed the feasibility and need for a large, multicentre RCT to test these PVC insertion models.

**Trial registration:**

Australian New Zealand Clinical Trials Registry, ACTRN12616001675415. Registered on 6 December 2016.

## Background

Peripheral intravenous catheter (PVC) insertion is the most commonly performed invasive procedure in hospitalised patients. Worldwide, it is estimated that 2 billion PVCs are sold each year [1] and used for the short-term delivery of intravenous (IV) medications and fluids [2]. However, multiple insertion attempts are common, and post-insertion failures from complications such as occlusion are as high as 69%, triggering the insertion of subsequent catheters [3–5].

PVC inserter models vary. Traditionally, intravenous therapy teams (IVTTs) were used for most PVC insertions [6, 7]. These teams were made up of nurses with advanced skills in insertion and maintenance [7, 8]. More recently, many IVTTs have been discontinued due to health care budget cuts, leaving PVC insertion to generalist nursing and medical staff [9]. Data supporting cost savings associated with disbanding IVTTs are yet to be reported in literature [9].

The generalist model involves nurses and medical staff at the clinical unit level [8], which ideally enables continuity of care if they are aware of the patient’s diagnosis and clinical history. A belief exists that even though generalists may have minimal PVC insertion skills, this rarely leads to negative outcomes [10]. This belief likely stems from a focus on PVC insertion success alone, but there is an ongoing concern that the varying skill level of generalist inserters leads to multiple needle sticks, patient discomfort and irreversible damage to the venous system, limiting current and future vascular access options [9, 11].

Other workforce models for PVC insertion include the use of vascular access specialists (VASs), who are practitioners with advanced assessment as well as technical skills for all vascular access devices. These practitioners may work within an IVTT [8] or within a specific unit or nursing framework [12] for device insertion, surveillance, research and education.

For the purpose of this trial, a VAS was defined as a clinician with advanced knowledge of vascular access, including catheter technology (materials and design); insertion assistive devices (such as ultrasonography); dressings; processes of catheter access; and management of IV therapy. This advanced level of expertise and knowledge is believed to preserve veins, enhance the patient experience, decrease the incidence of infusion complications and ultimately save on costs associated with clinician time, PVC-related products and length of hospital stay [12].

Effective PVC placement and the prevention of PVC-related complications are not only important clinical objectives but also essential for patient satisfaction [6]. A recent international survey exploring patients’ perspectives on PVC insertion found that consumers wanted standards implemented for inserters in order to feel safe and trust their health care professionals [13]. The level of skill of the PVC inserter is a risk factor for catheter failure [6, 14]. However, limited high-quality research exists exploring the efficacy of a VAS. Observational studies and audits report that VAS-inserted PVCs have fewer first-time insertion attempts [6, 15], less phlebitis [16, 17], less inflammation and catheter-related sepsis [18] and higher patient satisfaction [19]. As some of these studies were conducted before the year 2000 [16, 17, 20], it is unclear what impact more advanced PVC materials, dressings and other IV supportive equipment have had on PVC failure. Other observational studies have reported on the success of PVC insertion but not the subsequent catheter failure rate [6], or on the benefit of a VAS within a hospital but not compared to a generalist inserter [6, 7]. Limitations of previous research include data collected retrospectively [7], secondary analysis of existing datasets [14] or clinical staff assigning their own level of insertion skill [12].

There is a paucity of evidence from high-quality randomised control trials (RCTs) assessing inserter skill levels required for successful PVC insertion and prevention of device failure and complications. This makes it impossible for local and international guideline writers to produce comprehensive clinical practice guidelines for the best PVC insertion model of care. Therefore, it is important to examine the efficacy of different models for PVC insertion used in hospitals.

### The study

We compared standard care (generalist model: PVCs inserted in line with hospital policy by an accredited PVC inserter) with insertion by a VAS. The VAS for this pilot trial was a member of an intravenous therapy team for more than 20 years and an educator training clinicians to place PVCs in both a hospital and university program. The aim of this trial was to test the feasibility of conducting a suitably powered RCT by assessing both the methodology and rigour of methods planned for the larger study.

## Methods

### Study design and participants

We undertook a single-centre, parallel-group, pilot RCT in a large government teaching hospital in Queensland, Australia. Human research ethics committee approval was obtained from the hospital ethics committee (HREC/16/QRBW/386) and Griffith University (2016/782). The trial was registered with the Australian New Zealand Clinical Trials Registry (ACTRN12616001675415), and the protocol was published [21].

We recruited patients admitted to general medical and surgical wards between July and November, 2017. A research nurse (ReN) screened for patients who were over the age of 18, expected to have a PVC for greater than 24 h and able to provide written and informed consent. We excluded patients who had a current bloodstream infection or who had previously been enrolled in the study.

### Sample size

The recruitment target for this pilot RCT was 69 participants per group. This trial was not designed to have adequate power to detect statistical significances between groups, but rather to assess the feasibility of the methods to be used in a larger study. The sample size is considered appropriate for the purposes of feasibility assessment [22, 23].

### Randomisation and masking

The ReN obtained written informed consent and then, using a web-based central randomisation service (Griffith University Clinical Trials Randomisation Service, www151.griffith.edu.au), obtained group allocation, which was 1:1 with randomly varied small block sizes. Allocation was concealed prior to randomisation.

Two ReNs collected data for this trial. The first was responsible for recruitment and randomisation. The second ReN was masked to the study intervention and responsible for the daily PVC site inspections and device failure information. The endpoint of catheter-related bloodstream infection (CRBSI) was assessed by an infectious diseases physician who, along with the study statistician, was masked to group allocation. However, due to the nature of the study, blinding of patients and treating clinicians to the intervention received was not possible.

### PVC care and maintenance

All PVCs were inserted by hospital-accredited clinicians using local hospital policies. Skin decontamination for all insertions was with a 3M (St Paul, MN, USA) SoluPrep™ Antiseptic Swab (2% chlorhexidine gluconate [CHG] in 70% isopropyl alcohol [IPA]). All PVCs were Becton Dickinson (BD; Sandy, UT, USA) Insyte™ Autoguard™ Blood Control (non-winged) catheters with a Smart-Site™ Needle-Free Valve (BD) and a 10-cm extension tubing with a bonded three-way connector (Connecta™, BD). As per hospital policy, PVCs were to be re-sited every 72 h, unless the clinician chose to extend the dwell time in response to a clinical indication.

### Outcome measures

The primary outcome was to establish the feasibility of an adequately powered RCT using the following criteria: > 90% of patients screened would be eligible; > 90% of eligible patients would agree to enrol; > 90% of eligible patients would receive the allocated intervention; < 5% of enrolled patients would be lost to follow-up; and < 5% missing data.

The secondary outcomes included the following: (1) PVC failure, i.e. catheter removal before the end of therapy due to phlebitis (defined as two or more occurrences of pain, erythema, swelling, palpable cord or purulent discharge), infiltration (movement of IV fluids into the surrounding tissue), occlusion (PVC will not flush or leaks when flushed), accidental removal (partial or complete dislodgement of the PVC from the vein), infection (laboratory-confirmed local or PVC-related bloodstream infection [24]), PVC positive skin swab and/or positive PVC tip culture [25] (as per usual clinical practice); (2) PVC dwell time (from insertion until removal from either PVC failure, routine replacement or the completion of IV therapy); (3) insertion success; (4) insertion attempts; and (5) cost-effectiveness (a sub-set of PVC insertions observed and timed to establish estimates of staff costs and equipment).

### Data collection

Data for this study were collected by a ReN and entered into an electronic data platform supported by REDCap™ (Research Electronic Data Capture 6.10.6 © 2016 Vanderbilt University, Nashville, TN, USA) [26]. Feasibility outcomes (eligibility, recruitment, retention and attrition, protocol adherence and sample size estimates) were collected from enrolment screening logs.

At participant recruitment the ReN, who was also a VAS, collected patient demographic and clinical characteristics such as age, gender and vein quality as per the *peripheral vein assessment tool* [27]. From this assessment, taking into consideration the planned IV treatment and patient preference, the ReN/VAS documented their recommendation for vascular access device (VAD) choice and site selection. Participants were then randomised u. After the PVC was inserted, the ReN documented the gauge, profession of inserting clinician, number of insertion attempts, place of insertion and type of securement/dressing applied.

Trial participants were visited daily by the second ReN, who was masked to the intervention group. The ReN assessed patient satisfaction with the insertion procedure on an 11-point scale (0 = not satisfied and 10 = satisfied). The ReN also inspected the PVC site for redness, swelling and palpable cord (measured in centimetres from insertion site); patient-reported pain/tenderness (0 = no pain and 10 = maximum pain); leakage (yes/no); and purulence (none, from site, with ulceration). At PVC removal, the ReN recorded the date and time and the reason for removal. The participant was then asked to rate their overall satisfaction with the catheter on an 11-point scale (0 = not satisfied and 10 = satisfied).

### Statistical analysis

Feasibility outcomes were reported descriptively and analysed against predetermined acceptability criteria. Statistical analysis was performed using Stata 15 (Stata Corp, College Station, TX, USA). An intention-to-treat analysis framework was used; the unit of analysis was one PVC per patient. Missing data were not imputed. Frequencies and proportions were reported for categorical data. Mean values and standard deviations (SDs) were reported for normally distributed data; median values and 25th/75th percentiles reported otherwise. Covariates were re-categorised to suit the regression analyses as necessary and were not analysed in regression models if they had fewer than 20 cases. A graph of the Kaplan-Meier survival function was generated, and a log-rank test performed. Univariable and multivariable Cox regression was used to assess the effect of patient and treatment differences as well as for group comparisons. Covariates were deemed eligible for multivariable analysis at *p* < 0.20 and were dropped from the multivariable model during manual backward model building at *p* ≥ 0.05. The proportional hazards assumption was checked. *p* values < 0.05 were considered significant.

## Results

### Primary outcome

**Fig. 1:**
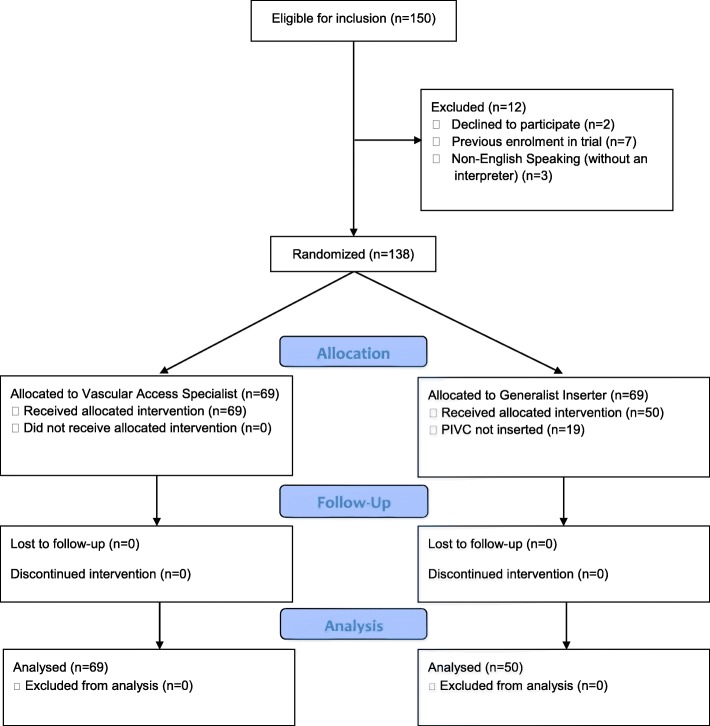
Consolidated Standards of Reporting Trials (CONSORT) flow chart

Between July and November 2017, 150 patients were screened, and 92% were eligible for trial recruitment. Willingness for study involvement was high, with only two patients declining trial participation. No patients were lost to follow-up, none received the incorrect study allocation and there were no missing outcome events; therefore, all predetermined feasibility criteria (Fig. 1) were met as per the trial protocol [21].

### Patient and PVC characteristics

At recruitment, patients had similar demographic characteristics between groups (Table 1). They were predominantly male, overweight or obese and admitted to a surgical ward. The 22 gauge PVC was more frequently used by VASs (67%) in comparison to generalists (50%). Generalists inserted more PVCs into the hand or wrist (46%) than the forearm (34%), whereas the VASs placed more catheters into the forearm (70%) than the hand or wrist (24%). The generalist inserters were medical staff (82%), anaesthetists (4%) and nurses (14%). The VAS inserter used ultrasound assistance with three insertions. Ultrasound was not used by the generalist group. Multiple insertion attempts occurred more often in the generalist (35%) than in the VAS group (19%).

**Table 1:** Descriptive statistics by study groups

	*n*	VAS	*n*	Generalist	Total
Group size^a^		69 (50)		69 (50)	138 (100)
Age (years)^b^	69	64.0 (47.0–73.0)	69	62.0 (47.0–71.0)	62.0 (47.0–73.0)
Sex: males	69	43 (62)	69	43 (62)	86 (62)
Weight category: overweight/obese	69	37 (54)	69	33 (48)	70 (51)
Skin integrity: good	69	41 (59)	69	36 (52)	77 (56)
Mobility at insertion:	69		69		
Independent		35 (51)		53 (77)	88 (64)
Required assistance to mobilise		21 (30)		9 (13)	30 (22)
Bed-bound		13 (19)		7 (10)	20 (14)
Reason for admission:	69		69		
Medical		19 (28)		19 (28)	38 (28)
Surgical emergent		14 (20)		21 (30)	35 (25)
Surgical elective		36 (52)		29 (42)	65 (47)
Infection at recruitment	69	21 (30)	69	21 (30)	42 (30)
Number of comorbidities:	69		69		
Zero		12 (17)		10 (14)	22 (16)
One		17 (25)		15 (22)	32 (23)
Two		11 (16)		17 (25)	28 (20)
Three		8 (12)		9 (13)	17 (12)
Four or more		21 (30)		18 (26)	39 (28)
Wound (present at recruitment)	69	42 (61)	69	40 (58)	82 (59)
Vein assessment:	69		69		
Excellent		18 (26)		25 (36)	43 (31)
Good		14 (20)		19 (28)	33 (24)
Fair or poor		37 (54)		25 (36)	62 (45)
Vein first choice for insertion:	69		69		
Cephalic		38 (55)		45 (65)	83 (60)
Medial antebrachial		15 (22)		6 (9)	21 (15)
Accessory cephalic		8 (12)		8 (12)	16 (16)
Other		8 (12)		10 (14)	18 (13)
Location first choice for insertion:	69		69		
Posterior lower forearm		32 (46)		45 (65)	77 (56)
Upper anterior forearm		20 (29)		7 (10)	27 (20)
Wrist		12 (17)		10 (14)	22 (16)
Other		5 (7)		7 (10)	12 (9)
Device sequence:	69		69		
Initial		3 (4)		0 (0)	3 (2)
Subsequent		66 (96)		69 (100)	135 (98)
Reason for insertion:	69		69		
IV medications only		20 (29)		20 (29)	40 (29)
IV medications and/or fluids		49 (71)		49 (71)	98 (71)
PVC is the appropriate device	69	58 (84)	69	55 (80)	113 (82)
Insertion difficulty (0 = none, 10 = max)^b^	69	2.0 (0.0–5.0)	11	2.0 (1.0–5.0)	2.0 (0.5–5.0)
Pain at insertion (0 = none, 10 = max)^b^	69	2.0 (1.0–3.0)	44	3.0 (1.0–4.0)	2.0 (1.0–3.0)
Device size (gauge):	69		50		
22		46 (67)		25 (50)	71 (60)
20		21 (30)		19 (38)	40 (34)
Other		2 (3)		2 (4)	4 (3)
Not documented		0 (0)		4 (8)	4 (3)
Reason for choosing size^c^:	69		11		
Clinician preference		41 (59)		10 (90)	51 (64)
Patient has limited vein size		33 (48)		2 (18)	35 (44)
Other		13 (19)		1 (9)	14 (18)
IV placement:	69		50		
Cephalic		31 (45)		20 (40)	51 (43)
Medial antebrachial		16 (23)		3 (6)	19 (16)
Accessory cephalic		10 (14)		3 (6)	13 (11)
Metacarpal		3 (4)		10 (20)	13 (11)
Other		9 (13)		14 (28)	23 (19)
IV location:	69		50		
Posterior lower forearm		26 (38)		13 (26)	39 (33)
Upper anterior forearm		22 (32)		4 (8)	26 (22)
Wrist		14 (20)		9 (18)	23 (19)
Hand		3 (4)		14 (28)	17 (14)
Other		4 (6)		10 (20)	14 (12)
Side of insertion: right	69	38 (55)	50	23 (46)	61 (51)
Skin hair prior to insertion:	69		50		
None present		31 (45)		31 (62)	62 (52)
Clipped		38 (55)		3 (6)	41 (34)
Unclipped		0 (0)		16 (32)	16 (13)

Frequencies and column percentages shown, unless otherwise noted

^a^ Row percentages shown

^b^ Median and 25th–75th percentiles shown

^c^ Multiple responses allowed

*n* number of non-missing observations, *VAS* vascular access specialist, *IV* intravenous, *PVC* peripheral intravenous catheter, *max* maximum

The initial masked vein assessment identified a higher number of participants with fair or poor veins randomised to the VAS insertion group (54%) compared with 36% in the generalist group. The ideal site and vein for PVC placement assessed by the VAS prior to randomisation was achieved for 81% of PVCs placed by the VAS, compared with 26% of the generalist inserters.

### Secondary outcomes

All PVCs (*n* = 69) were successfully inserted in the VAS group. In the generalist group, 19 (28%) patients did not have a PVC inserted and, in response, were changed to oral medication (*n* = 8), had a pre-existing PVC left in place (*n* = 6), had a peripherally inserted peripheral catheter inserted (*n* = 2) or remained waiting for a PVC insertion for at least 24 h (*n* = 3).

PVC insertion timings and procedural resource usage were collected for 16 VAS and 4 generalist insertions. The mean PVC insertion procedure time was 2 min in the VAS group and 11 min in the generalist group. A full cost-effectiveness analysis could not be undertaken due to limited generalist group data collected as a result of the following: (1) long delays from PVC request to insertion of the catheter; (2) the fact that many patients in the generalist group did not ultimately have a PVC inserted.

**Fig. 2:**
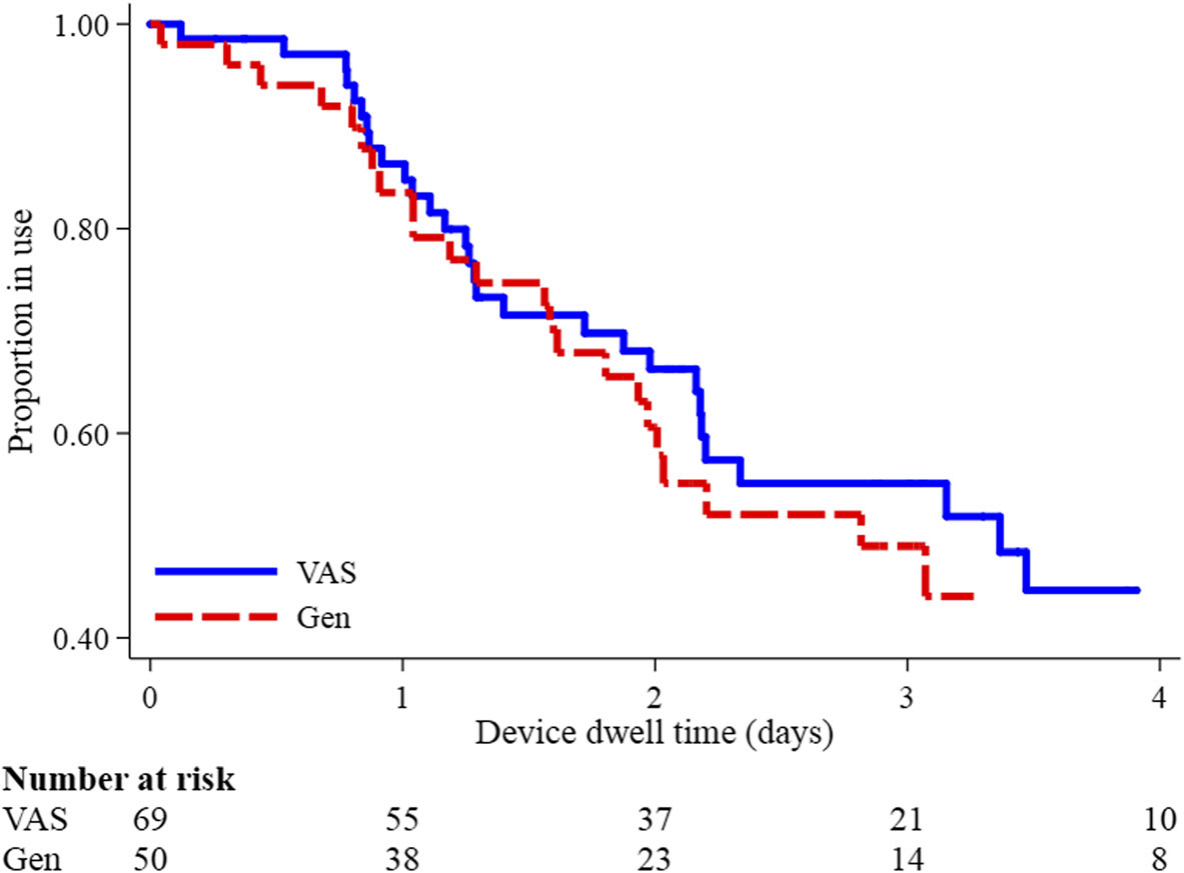
Kaplan-Meier survival analysis

PVC post-insertion failure was 54% in the generalist group and 48% in the VAS group (Table 2). This equated to 217 and 228 failures per 1000 PIV days respectively (incidence rate ratio 1.05, 95% confidence interval [CI] 0.61–1.80, Table 2); these results were not different upon Kaplan-Meier survival analysis (Fig. 2; log-rank *p* = 0.92). The most common causes of failure were phlebitis and infiltration. Even though this study was not powered to show effect, phlebitis was 5% higher in VAS-inserted catheters than for generalist insertions. Occlusion and partial or complete dislodgement were higher (absolute 8% and 5% respectively) in generalist-inserted PVCs. There were no local or PVC-related bloodstream infections in either group.

**Table 2:** Study outcomes (*n* = 119)

	VAS	Generalist	*p* value
	*n* = 69	*n* = 50	
PVC successfully inserted	69 (100)	50 (72)	
Multiple insertion attempts^a^	13 (19)	16 (35)	
Number of insertion attempts^a, b^	1.22	1.74	
Reason for removal:			
Treatment complete without complication	29 (42)	19 (38)	
Treatment incomplete with complication	26 (38)	22 (44)	
Treatment completed with complication	7 (10)	5 (10)	
Routine re-site or theatre replacement	5 (7)	3 (6)	
Insertion of a CVAD	2 (3)	1 (2)	
Device failed	33 (48)	27 (54)	0.506^c^
Positive blood count	0 (0)	2 (4)	
Complication^d^:			
Phlebitis	19 (28)	10 (20)	
Infiltration	13 (19)	9 (18)	
Occlusion	7 (10)	9 (18)	
Accidental removal	6 (9)	7 (14)	
Unknown	0 (0)	1 (2)	
Device days	152	118	
Incidence rate of failure^e, f^	217 (154–305)	228 (156–332)	
Incidence rate ratio	Reference	1.05 (0.61–1.80)	0.924^g^
Overall patient satisfaction^h, i^			
Insertion	9 (8–10)	7 (3.5–9)	
Overall	7 (6–9)	4.5 (1.5–6)	

Frequencies and column percentages shown, unless otherwise noted

^a^Successfully inserted devices only

^b^Average shown

^c^Chi-squared test

^d^Multiple responses allowed

^e^Per 1000 device days

^f^Includes 95% confidence interval

^g^Log-rank test

^h^Median (25th/75th percentiles) shown

^i^0 = not satisfied, 10 = satisfied

*VAS* vascular access specialist, *n* number of non-missing observations, *PVC* peripheral intravenous catheter, *CVAD* central venous access device

Median satisfaction with PVC insertion was higher in the VAS group (9 versus 7) than the generalist group (Table 2). Overall median satisfaction with the PVC was also higher in the VAS group (7 versus 4.5, Table 2) than in the generalist group.

### Multivariable modelling for PVC post-insertion failure

Although this study was not powered to show statistical significances between groups, in the multivariable model (Table 3), medical diagnosis (*p* < 0.001) or bed-bound status at insertion (*p* < 0.05) were associated with an approximately twofold higher incidence of PVC failure, and non-sterile tape securement remained associated with decreased PVC failure (hazard ratio [HR] 0.36, 95% CI 0.18–0.70, *p* < 0.001).

**Table 3:** Cox regression

	Univariable	Multivariable *n* = 119
Study group: generalist (ref. VAS)	1.03 (0.61–1.73)	1.18 (0.70–2.00)
Sex: male (ref. female)	0.61 (0.35–1.05)*	#
Age (increment of 1 year)	1.00 (0.98–1.01)	^
Body mass index (increment of 1)	0.99 (0.94–1.03)	^
Comorbidities (none /1/2/3/4 or more)	0.96 (0.81–1.16)	^
Insertion on dominant side (ref. no)	1.03 (0.62–1.72)	^
Bed-bound at insertion (ref. no)	1.74 (0.93–3.24)*	2.17 (1.14–4.11)**
Medical reason for admission (ref. surgical)	2.08 (1.22–3.55)***	2.08 (1.21–3.57)***
Infection at recruitment (ref. no)	0.90 (0.52–1.55)	^
Vein assessment (ref. excellent):		^
Good	0.70 (0.32–1.52)	^
Fair/poor	0.34 (0.67–2.10)	^
Location selected was location in which PVC was placed (ref. no)	0.79 (0.47–1.34)	^
Difficulty with previous insertion (ref. no)	0.99 (0.56–1.74)	^
PVC is the appropriate device (ref. no)	0.72 (0.39–1.32)	^
Device gauge: other (ref. 22)	0.64 (0.36–1.11)*	#
Location (ref. posterior lower forearm):		^
Upper anterior forearm	1.49 (0.75–2.97)	
Wrist	1.31 (0.61–2.79)	
Hand	1.89 (0.87–4.14)*	
Other	0.73 (0.27–1.98)	
Multiple insertion attempts (ref. no)	0.95 (0.53–1.73)	^
Dressing: non-sterile tape^a^ (ref. never)	0.38 (0.20–0.74)***	0.36 (0.18–0.70)***
Dressing: Tubigrip^a^ (ref. never)	0.81 (0.46–1.42)	^
Dressing dirty/wet/damaged^a^ (ref. never)	0.89 (0.42–1.85)	^
Fluids ^a^ (ref. never)	1.00 (0.59–1.68)	^
Antibiotics^a, b^ (ref. never)	1.35 (0.72–2.51)	^
Anaesthesia^a^ (ref. never)	0.78 (0.42–1.46)	^
Cefazolin^a^ (ref. never)	0.87 (0.47–1.62)	^
Pain relief ^a^ (ref. never)	0.97 (0.56–1.66)	^
Other IV medication^a, c^ (ref. never)	0.71 (0.38–1.31)	^
Antiemetic and antireflux ^a^ (ref. never)	0.93 (0.50–1.73)	^
Accesses (total, none /1 to 3/4 to 6/7 or more)	0.85 (0.69–1.05)*	#

Hazard ratios and 95% confidence intervals shown

*p value < 0.20, **p value < 0.05, ***p value < 0.01

*#* dropped from multivariable model at *p* ≥ 0.05, *^* ineligible for multivariable analysis at overall *p* ≥ 0.20

^a^At any time during study

^b^Includes ampicillin, benzylpenicillin, gentamicin, vancomycin, ceftazidime, azithromycin, meropenem, cefepime, or augmentin

^c^Includes Frusemide, contrast, insulin, magnesium, or thiamine

*VAS* vascular access specialist, *PVC* peripheral intravenous catheter

## Discussion

Improving the knowledge and skill of PVC inserters is likely to reduce the current situation where patients commonly experience multiple PVC insertion attempts and unacceptably high post-insertion catheter failure rates. In this pilot RCT, we compared two inserter workforce models for clinical, patient and feasibility outcomes. All predetermined feasibility outcomes were met; thus, we have established that the tested methods are appropriate for an adequately powered, multicentre RCT. Overall PVC failure was higher in the generalist compared with the VAS group, and although pilot trials are not powered for statistical significance, this result was clinically meaningful and needs testing in a larger RCT. To compare 54% versus 48% post-insertion failure with 80% power (*p* = 0.05) would require 1084 patients per group (powerandsamplesize.com).

Under generalist models, establishment of vascular access is frequently left to junior medical and nursing staff who, with minimal knowledge about complications associated with IV medications, may choose a PVC as a default VAD [27, 28]. In our trial, 18% of PVCs were considered an inappropriate VAD by our blinded VAS assessor, as they had IV therapy prescribed for greater than 5 days and/or poor vascular access. Generalist models also lack standardisation of knowledge and technique for clinicians inserting PVCs, meaning that expertise and maintenance of competence cannot be guaranteed across and within health care settings [29]. Such deficiencies likely contributed to the lower first-time PVC insertion success in our generalist group, with multiple insertion attempts occurring almost twice as often with the generalist inserter (35%) than the VAS inserter (19%). An additional three patients allocated to the generalist group were still awaiting PVC placement after 24 h and numerous unpleasant insertion attempts. This is not only a poor patient experience but also has cost implications for clinician time, delayed treatment and potentially extended hospital stay.

More than a quarter of patients allocated to the generalist group in our trial did not receive a PVC, compared to 100% placement by the VAS group. The purpose of placing a PVC is to start or continue treatment, and this was an unexpected result. It may indicate a lack of PVC insertion skill in the generalist group or a lack of comprehensive assessment regarding the requirement for a PVC. Either way we consider that this may be the more appropriate primary endpoint for a follow-on study. The most commonly reported reason in this study for non-placement was the change of antibiotic therapy from intravenous to oral. The decision to change antibiotic route should be based on three key factors: (1) the antimicrobial agent, (2) the patient and (3) the condition being treated, and careful consideration is necessary to provide the best care [30]. Within this trial it was unclear if these factors were the determining considerations, or if this change was due to the unavailability of staff to place the catheter or unsuccessful insertion attempts rather than the patient’s clinical need.

Our multivariable model identified that patients admitted with a medical diagnosis and those who were non-ambulant (bed-bound) were at higher risk of PVC failure. These patients may have a higher risk, as they are likely to have more comorbidities and a history of greater VAD use than ambulant patients and/or those with an acute surgical diagnosis. These patients should be the priority for efforts to improve PVC outcomes. We also found that patients whose PVC was secured with additional non-sterile tape had a significant association with decreased PVC failure. These results reflect a similar finding by a large cohort study at the same hospital that reported significantly lower occlusion/infiltration, phlebitis and dislodgement rates when non-sterile tape was used as an additional PVC securement [31]. This suggests that, despite advances in dressings and securement devices, PVC failure rates have remained high, and even good-quality insertion requires effective securement to maintain function.

### Limitations

The main limitation of this study is that it is a pilot RCT, and although the results are clinically interesting, the study is not designed to provide definitive conclusions about the best model for PVC insertion. The use of the same VAS to perform the pre-randomisation assessment and to insert the PVCs in the VAS group was also a limitation of this study. However, the VAS was blinded to allocation at the time the assessment was conducted, and information was directly entered into an electronic platform at the bedside.

## Conclusion

This study suggests that less insertion failure and less post-insertion failure occur when catheters are placed by a VAS. This pilot trial has confirmed the feasibility and clinical need for a large, multicentre RCT to test these PVC insertion models to provide evidence for health service delivery improvements.

## Abbreviations

CRBSI: Catheter-related bloodstream infection
HR: Hazard ratio
IV: Intravenous
IVTT: Intravenous therapy team
PVC: Peripheral intravenous catheter
RCT: Randomised controlled trial
ReN: Research nurse
SD: Standard deviation
VAS: Vascular access specialist
